# Increasing vaccination intention in pandemic times: a social marketing perspective

**DOI:** 10.1007/s43039-022-00049-w

**Published:** 2022-01-31

**Authors:** Philipp Wassler, Giacomo Del Chiappa, Thi Hong Hai Nguyen, Giancarlo Fedeli, Nigel L. Williams

**Affiliations:** 1 Department of Management, University of Bergamo, Via dei Caniana, 2, 24127 Bergamo, Italy; 2 Department of Economics and Business, University of Sassari, Via Muroni, 25, 07100 Sassari, Italy; 3 Department of Marketing, Events and Tourism, Business School, University of Greenwich, Old Royal Naval College, Park Row, London, SE10 9LS United Kingdom; 4 Moffat Centre for Travel and Tourism Business Development, Glasgow Caledonian University, Cowcaddens Rd, Glasgow, G4 0BA United Kingdom; 5 Operations and Systems Management, University of Portsmouth, Winston Churchill Ave, Southsea, Portsmouth, PO1 2UP United Kingdom

**Keywords:** Social marketing, COVID-19 vaccines, Vaccine marketing, Vaccination intention, Italy, Travel craving

## Abstract

With the release of several COVID-19 vaccines, hopes for ending the pandemic have emerged. However, the uptake of the vaccines is largely voluntary and depends on the intentions of the public. From a social marketing perspective, this study takes the case of Italy to identify and test factors that are likely to increase COVID-19-vaccine intention. A sample of 3893 respondents was collected throughout Italy and a model empirically tested by structural equation modeling. The findings suggest that a social marketing campaign for undertaking COVID-19 vaccines should educate the public, going beyond just safety and efficacy, and create positive social norms by combatting misinformation on various platforms, including social media. Furthermore, it was found that economic hardship from COVID-19 does not automatically translate to vaccination intention and that social marketing campaigns should particularly target economically vulnerable and important segments. Also, instilling a craving for travel could potentially stimulate citizens to undertake COVID-19 inoculation. Finally, contributions and implications for social marketing COVID-19 vaccines in Italy and elsewhere are addressed.

## Introduction

The COVID-19 vaccines were one of the most eagerly awaited medicines in modern history (Cavaleri et al., 2021). Approximately 9 months after the initial outbreak of the pandemic, the European Medicines Agency (EMA) recommended the first EU conditional marketing authorization (CMA) for the BioNTech mRNA vaccine (European Medicines Agency, 2020). CMA is a response to public health threats used in emergencies by the European Union and is based on a demonstration of a positive benefit-risk balance and comes with additional obligations, such as further studies to ensure the pharmaceutical quality of the vaccines.

However, most countries and communities have faced groups or individuals which are delaying or refusing the vaccine uptake. Nowak, Gelling, MacDonald, Butler, and the SAGE Working Group on Vaccine Hesitancy [EMA] (2015) underline that it is crucial to highlight the factors which influence vaccination intention in this situation. Previous attempts have shown that there is no single intervention strategy to solve this problem (e.g., Dubé et al., 2014; Dubé et al., 2015; Larson et al., 2014).

Generally, social marketing has been recognized as a valuable tool to advocate these immunization programs (Butler & McDonald, 2014; Opel et al., 2009). Nowak et al. (2015) highlight that social marketing programs have mainly focussed on increasing awareness and addressing questions and concerns to motivate behavior. Evans and French (2021) as well as French, Deshpande et al. (2020) also point out that this is likely local context and population specific. What these exact social marketing practices should be based on for the COVID-19 pandemic is not yet fully understood.

Social marketing represents an extension of traditional marketing principles and refers to the “selling” of ideas, attitudes, and behaviors, which are usually characterized as “pro-social” or focussed on improving the health or wellbeing of targeted individuals or the society (Lee & Kotler, 2019; Lefebvre, 2013; Suarez-Almazor, 2011). The difference to traditional marketing thus lies in the fact that social marketing does not encourage consumers to purchase branded products or services but leads individuals to adopt certain ideas or recommended actions (Nowak et al., 2015). Related to vaccination intentions, the question is thus how social marketing can “nudge” an individual or population towards vaccine uptake (French & Gordon, 2020). This would thus necessitate the identification of determinate “key factors” which are likely to stimulate vaccine-related behavior positively in certain populations. In other words, there is a strong need to understand different types of drivers to inform community-led communication strategies to build trust and optimize COVID-19 vaccine uptake (Rhodes, Hoq, Measey, & Danchin, 2020, p. 110). Although no key factors for social marketing campaigns targeting the COVID-19 vaccine uptake are agreed upon, several potential trends can be identified from the relevant literature.

This paper focuses on three macro factors (vaccine, media, and travel-related), based on the three concepts of direct perceptions of the vaccines, sources of information, and the need to resume leisure and economic activities which have been severely limited by the pandemic. Studies have shown that questioning the safety and efficacy of the available vaccines is a potentially major barrier to their uptake (Bish et al., 2011; Larson, 2018; Larson et al., 2011; Larson et al., 2016; Yaqub et al., 2014). Media-related factors are also likely to play a significant role in this context, as the COVID-19 pandemic has been labelled as the first “misinfodemic” and media outlets have questioned the safety and efficacy of the vaccines (Williams et al., 2020), often spreading misinformation. In some cases, vaccine-resistant communities have even used celebrities and influencers to promote resistance online (Puri et al, 2020). Evans and French (2021) also highlight that the Center of Countering Digital Hate had tracked over 400 anti-vaccine accounts over social media platforms in March 2021, with over 60 million followers and people joining daily. Accordingly, groups running these accounts were sometimes entrepreneurs advertising homoeopathic cures for immunization or conspiracy theorists who profit from online advertising revenue. The last factor considered is related to economic dependency and its relationship to vaccine uptake. The tourism industry is chosen as the pandemic has crippled the global tourism industry in a particularly hard way. There is an ongoing struggle with borders being closed, bans on visas for certain nationalities, and airports turning into parking lots (Abdullah, 2020; Scott, 2020). The World Travel and Tourism Council (2020) stated that the pandemic could affect up to 50 million jobs in the tourism industry worldwide, with Asia being likely the most affected continent and an expected 10 months or longer recovery time after COVID-19 eventually comes to an end. There are thus potential interests from the tourism demand and supply side to get vaccinated. From the demand side travel craving and intentions might lead to vaccine uptake, particularly since the release of the European Green Pass in July 2021. From the supply side, dependency on the tourism industry might lead potential hosts to uptake the vaccine for financial needs. Factors directly related to the vaccines, factors related to media, and factors related to economic activities (in this case travel) are hypothesized to potentially influence vaccine uptake.

This study will thus provide insights into how social marketers may adapt their work practices finalised to improving vaccine uptake, taking the case of Italy to empirically test social marketing-related factors which are likely to increase COVID-19-vaccination intention. At the time of writing, Italy has achieved significant progress with its vaccination campaign, with almost 50% of the population having completed the vaccination cycle in late July 2021 (Governo.it, 2021). However, specific population subsets have shown resistance against undertaking the vaccine with unvaccinated health workers facing suspensions (ANSA, 2021). Therefore, while Italy represents an appropriate case study where vaccines are widely available and have been extensively distributed, certain subsets of the population still display low intention for undertaking COVID-19 vaccines. We take the case of 3839 respondents to Italy to identify and test factors that are likely to increase COVID-19-vaccine intention through Structural Equation Modeling. This paper thus tests the aforementioned factors in this context and provides insights into how social marketers can potentially enhance vaccine uptake in Italy and, possibly, elsewhere. The findings show that social marketing campaigns for undertaking COVID-19 vaccines should educate the public, going beyond just safety and efficacy, and create positive social norms by combatting misinformation on various platforms. We also show that social marketing campaigns should particularly target economically vulnerable and important segments and that the reprisal of non-essential activities that have been limited by the pandemic can also stimulate vaccine uptake.

## Theoretical development

### Vaccination intention and Covid-19

Individuals and groups can display a range of intentions related to vaccines, ranging from vaccine promotion to refusal (Dubé, Laberge, Guay, Bramadat, Roy & Bettinger, 2013). This spectrum incorporates individuals who may display subsequent behaviors such as refusal of specific vaccines, delay vaccines or accepting vaccines reluctantly. The intention to undertake COVID-19 vaccine for the general population(s) is and will be a major challenge for governing bodies throughout and even in a post-crisis context (Detoc, Bruel, Frappe, Tardy, Botelho-Nevers, & Gagneux-Brunon, 2020). Rhodes et al. (2020) that COVID-19 vaccination intention can likely be stimulated by identifying certain key factors or triggers, which can then be used in a social marketing campaign.

Several theories give an insight into what might motivate people to undertake COVID-19 vaccines. Social Exchange Theory has frequently been used to investigate motivation and behavior. Essentially, Social Exchange Theory studies the interaction of two parties and their perceived risks and benefits implementing a cost-benefit analysis (Dutilh Novaes, 2020). In this case, it could thus be assumed that if the benefits outweigh the risks, an individual would be willing to uptake a COVID-19 vaccine. This could be related to general trust in the vaccines or activities which are not possible to pursue without taking the vaccines. Although this theory is important to identify what might inspire vaccine-related behavior, Social Exchange presumes that human reasoning is fully rational and is not always sufficient to explain irrational beliefs and values which are not grounded in personal experience (Wassler et al., 2019). These can be explained through Social Representations Theory, investigating a set of ideas, values, knowledge, and explanations forming the social reality of a community (Moscovici, 1988) rather than just a personal cost-benefit analysis. This study will consider both, rational social exchange principles based on the cost-benefit analysis of an individual and social representations, beliefs that are formed and shared by the community.

More specifically, this paper focuses on three macro factors (vaccine-related, media-related, and travel-related), based on the three concepts of direct perceptions of the vaccines, sources of information, and the need to resume leisure and economic activities which have been limited by the pandemic. While direct perceptions of vaccines and the relevant sources of information have been investigated in several (pre-pandemic and pandemic) contexts (e.g., Malik, McFadden, Elharake, & Omer, 2020; Mannan & Farhana, 2020), the reprisal of activities limited by the pandemic as a motivator for uptaking the vaccine has only rarely been considered. Recent data has shown that the introduction of “vaccine passports” has motivated vaccine uptakes, such as the case of Italy where the “European Green Pass” is needed for entering restaurants, workplaces, and long-haul transports (Il Sole 24 Ore, 2021). Travel and tourism, if not related to medical urgencies or work, is usually considered non-essential activity. While data has shown increasing vaccine uptake to undertake essential activities such as accessing the workplace, traveling constitutes an interesting factor of non-essential activities which might motivate vaccine uptake. The chosen factors will be discussed more in detail as follows.

### Factors leading to vaccination intentions and hypotheses

#### Vaccine related factors

Vaccination intention is commonly influenced by factors of knowledge, experience, perception, and group influences. These factors are often related to confidence in a determined vaccine, which commonly refers to a wide concept of trust related to vaccines or a specific vaccine (Harrison & Wu, 2020). The behavior which is the result of the confidence in a vaccine can thus range anywhere from vaccine advocacy to vaccine refusal (Dubé et al., 2013). It is anyhow important to notice, that low vaccine confidence does not always relate to outright refusal, but also to delaying vaccines or reluctant acceptance. These individuals are often referred to as “hesitant compliers”, such as worried parents getting vaccines for their children for community benefit rather than for personal effort (Enkel, Attwell, Snelling, & Christian, 2018). Cafiero, Guille-Escuret, and Ward (2021) add that in addition to refusal, individuals with low vaccine confidence often question the novelty, ingredients, or timing of specific vaccines. This can also vary by vaccine type, and general vaccine confidence would not automatically translate to individuals up taking a specific vaccine (Little, Goodridge, Lewis, Lingard, Din, Tidley, Roberts, Williams, & Hayes, 2015), such as the COVID-19 vaccines. However, if an individual does feel confident in a certain vaccine, more positive attitudes towards vaccine uptake are likely. The following hypothesis is thus proposed:

##### H1

COVID-19 vaccine confidence is likely to influence COVID-19 vaccination intention positively

Adongo, Amenumey, Kumi-Kyereme, & Dubé (2021) highlight that vaccination intention is likely related to vaccine concerns, with the most salient being vaccine efficacy and vaccine safety. These two factors of concern are particularly important for the COVID-19 vaccines on the market (Williams et al. 2020).

According to Yaqub et al. (2014), vaccine efficacy concerns refer to doubts that will function or perform to the level expected. Cases for vaccine efficacy concerns have been shown in past studies and real-life examples. Specific to the Muslim Hajj pilgrimage, Bish, Yardley, Nicoll, and Michie (2011) demonstrate a low uptake of influenza vaccine, which was particularly related to low trust in vaccine efficacy among the pilgrims. Specific to COVID-19, polemic voices have been raised over presumed low levels of efficacy in certain vaccines (often Sinovax or Sputnik V) and on vaccinated individuals still being infected with the virus. Often anyhow, a lower death rate among fully vaccinated people has been ignored by efficacy skeptics.

Safety concerns, on the other hand, refer to worries that up-taking a vaccine might lead to harmful outcomes (Yaqub et al., 2014). These beliefs are often propagated by vaccine skeptics, conspiracy theorists, and anti-vaxxer activists (Kata, 2012). Among the most common beliefs is that vaccines include toxic ingredients, cause unrelated diseases and conditions (e.g., autism), or the belief that they cause the disease which they are meant to protect from. Safety concerns have shown to be a major issue with the COVID-19 vaccine campaigns. In Italy, several cases of thrombosis were linked to the Vaxzevria vaccines and although the risk factor is extremely low, this has led to a lower vaccine uptake in certain regions and eventually governments suspending this type of vaccine for certain age groups or the general population. General safety concerns about the COVID-19 vaccines are often related to the relatively short periods in which they have been developed.

Often these factors have been propagated by political parties or activists, evangelizing their beliefs, and attempting to capitalize on vaccine scares (Ward et al., 2019). The following hypothesis is thus developed:

##### H2

COVID-19 vaccine concerns (efficacy and safety) are likely to influence COVID-19 vaccination intention negatively

#### Social media

The modern world highly relies on information that is constantly generated, shared, and consumed by product and service providers and users worldwide. While social media is a ubiquitous phenomenon, the intensity of how its information is being used by individuals varies highly. Fedeli (2020) highlighted an increased risk of manipulation of information on social media, that may shape differently to the benefit or detriment of certain entities or organizations. As content shared on social media varies highly in reliability and trustworthiness, users who have established larger social networks or show a higher intensity of use overall, are more likely to be influenced by social media content (Kaiser, Keller, & Kleinen-von Königslöw, 2018). This is particularly relevant for the COVID-19 pandemic, where an unprecedented amount of content has been shared on social media, and users might come across conflicting information about the vaccines. Studies have shown that most of the COVID-19 related misinformation is spread through social media and that social media users are more likely to be exposed to them (e.g. Apuke & Omar, 2021; Srivastava et al., 2020; Yang & Tian, 2021). In other words, social media users are more likely to come across false information on the vaccines than individuals who rely more on official or traditional media. Although the debate about vaccines on social media also includes vaccine-supporters and official sources, studies have shown that there is a disproportionate amount of anti-vaccine misinformation being spread through social networks (Germani & Biller-Andorno, 2021; Jennings et al., 2021; Piedrahita-Valdés et al., 2021). As individuals are influenced by their social media use, one might expect that a negative attitude towards vaccine might thus be stimulated. The following hypothesis is thus proposed:

##### H3

Social Media Intensity are likely to influence COVID-19 vaccination intention negatively

Social media are frequently related to misinformation and in the case of the current pandemic, this has exceeded unprecedented levels. As a result, Williams et al. (2020) have labeled it a “(mis)infodemic”, where actors have purposefully (to different extents) spread fake news about the pandemic and vaccines online. Notable cases include the initially exaggerated effects of hydroxychloroquine on the infection, vaccines containing microchips, 5G towers spreading the virus further, and other politically and ideologically motivated conspiracy theories. With the advent of several vaccines, negative misinformation spread online has also shown direct impacts on the vaccination campaign, with initial news about thrombosis risks of Vaxzevria being exaggerated in Italian media and vaccine uptakes falling dramatically in the subsequent weeks. Often, this misinformation has been used by anti-vaxx and other fringe groups (such as far-right activists) to wilfully create upheaval. Studies across different continents have also shown that fake news about the available vaccines were largely negative and were related to hesitancy about inoculation (Talabi et al., 2021; Tawat, 2021; Kanozia & Arya, 2021; Lyu et al., 2021). The following hypothesis is thus proposed as follows:

##### H4

COVID-19 Social Media Misinformation is likely to influence COVID-19 vaccination intention negatively

#### Travel and tourism

The tourism industry has been one of the hardest hit by the ongoing pandemic. However, recent research shows the rise of trends such as “revenge tourism”, i.e., tourists making up for the time lost by traveling even more once the pandemic allows doing so (Wassler & Fan, 2021). Travel intention refers to the likelihood and commitment towards the idea to travel, and it is influenced by attitudinal and practical factors (Sönmez & Graefe, 1998). In this case, the practical factors are largely represented by the structural limitations that COVID-19 has caused for international and domestic travel, with borders being closed, quarantines put in place, and even free movement within certain countries being restricted. With the introduction of the European “Green Pass” in summer 2021 and vaccination campaigns going ahead on a global scale, some potential tourists would likely see the vaccine as a way to mitigate some of their travel limitations. The following hypothesis is thus proposed:

##### H5

Travel Intention (domestic and international) is likely to influence COVID-19 vaccination intention positively

Travel craving comes from clinical psychology and usually refers to extremely liking of, or compulsory use of a substance (Kavanagh et al., 2013). Mitev and Irimiás (2021, p.2) define the concept in a travel context as “a travel-focused cognitive-emotional event with aversive or incentive properties experienced when a person who wishes to travel cannot do so, for reasons beyond their control.” Contrary to travel intention, the travel craving construct refers to a desire for a certain experience that helps to cope with restrictions in difficult times (Drummond, 2001) and is not necessarily a precursor to an actual trip (Mitev & Irimiás, 2021). Further, typical factors deemed relevant for the understanding of travel decision making (ie. both emotional and practical) may ascertain their limitations of predictability amidst the peculiarity of the COVID-19 scenario. Given the strong relevance of uncertainty that comes with the prospects of traveling during the pandemic and the reasons presented above, travel craving may may prove particularly relevant in the context presented. The following hypothesis is therefore proposed:

##### H6

Travel craving is likely to influence COVID-19 vaccination intention positively

The economic impact of the COVID-19 pandemic on the tourism industry has been unprecedented in recent times (Newsome, 2020; Sigala, 2020; Ugur & Akbıyık, 2020) and financial losses have been dramatic. The dependency on the tourism industry is important not only on a national but also on an individual level. Studies have found that economic dependency is a key factor in forming positive attitudes towards the tourism industry in general (Jurowski et al., 1997; McGehee & Andereck, 2004). It is also widely believed that the key to reviving the tourism industry is successful vaccination campaigns across different countries (Williams et al., 2020). It can thus be assumed, that individuals who depend financially on the tourism industry would be more willing to uptake the vaccine which will ultimately aid the revival of the industry. This hypothesis is presented as follows:

##### H7

Tourism Dependency is likely to influence COVID-19 vaccination intention positively

## Methodology

### Data collection

The survey instrument includes the measurement scales of Vaccination Intention and its antecedents with a 5-point Likert scale (including COVID-19 Vaccine Confidence, COVID-19 Vaccine Concerns, Social Media Intensity, COVID-19 Misinformation, Travel Intention, Travel Craving and Tourism Dependency) as well as social demographic characteristics. COVID-19 Vaccination Intention was measured by the likelihood of taking the COVID-19 vaccines. Shapiro et al.'s (2018) vaccine hesitancy scale was adapted to measure COVID-19 Vaccine Confidence, comprising of 8 measurement items. COVID-19 Vaccine Concerns was considered as a formative higher-order construct, consisting of 2 lower-order components of Efficacy Concerns and Safety Concerns, measured by 10 items which were adapted from Adongo, Amenumey, Kumi-Kyereme, and Dub'e (2021). Covid-19 misinformation measure was adopted from Williams et al. (2021). Travel Intention was also a formative higher-order construct with 2 lower-order components of Domestic Travel Intention and International Travel Intention, each was measured by 3 items. Social Media Intensity scale, containing 6 items, was adopted from Ellison, Steinfield, Lampe (2007). Travel Craving were measured by 5 items adapted from Mitev and Irimiás (2020), as well as Covid-19 misinformation (Williams et al. 2021). The full list of measurement items is provided in Appendix 3.

Data for this study were collected from Italians using an online self-administrated questionnaire utilizing a snowball sampling technique. Through referral mechanisms, this technique allows reaching an ever-expanding number of potential respondents inexpensively and efficiently (Goldenberg et al. 2009). Even though it is not considered a random sampling approach, it was considered as the best sampling approach for this study because it allowed the research team to obtain data from a large sample of consumers across different regions in Italy (including those from remote areas). Moreover, it was also considered to be appropriate to cope with the financial constraints of this project (Stevens, Loudon & Wrenn, 2006) as well as with the social distancing rules and travel restrictions, especially across regions, imposed by the Italian government.

The initial subjects were generated via a panel of 2000 Italians residing in different Italian regions. The process was also supported by MANAGERITALIA, an Italian Association that supported the data collection by inviting their members (i.e. managers of different ages working in the service sector and residing all over Italy) to take part in the survey. All the individuals receiving the email invitation were also encouraged to forward the survey to their friends and acquaintances. The data collection was carried out from 25th January to 15th February 2021. A total of 4020 responses were obtained. However, 157 responses were eliminated due to a high percentage of missing data. Finally, a total sample of 3893 was used for further analyses.

The sample profile is presented in Table 1. Within the sample, the number of male respondents was slightly higher than the female ones, with 57.4% and 41.8% respectively. It was distributed across the age groups, with about 13% to 23% of respondents in each group, except 3.1% of the youngest group, i.e. 18–25. In terms of education, most of the respondents had completed high schools (34.2%) and/or colleges/universities (45.7%). About a fifth of the respondents (20.7%) did not disclose their income and about a third (33.8%) of those who did, indicated a high level of the annual income of 50,000 EUR or more. Almost half of the respondents were claimed to be financially dependent on tourism to various extents, from slightly to extremely. The sample was also distributed across different regions of Italy, of which North-West and South, received the highest numbers of respondents, i.e. 37.5% and 28.4% respectively, followed by the North-East (13.7%), the Centre (12.1) and Islands (7.09%).

**Table 1:** Respondent profile

	Total (3893)	
	N	(% or SD)
*Gender*		
Male	2236	(57.40)
Female	1627	(41.80)
Missing	30	(0.80)
*Age*		
18–24	119	(3.10)
25–34	490	(12.60)
35–44	644	(16.50)
45–54	899	(23.10)
55–64	910	(23.40)
Above 65	821	(21.10)
Missing	10	(0.30)
*Education*		
Primary school	2	(0.10)
Secondary school	97	(2.50)
High school	1330	(34.20)
College/university	1778	(45.70)
Postgraduate	675	(17.30)
Missing	11	(0.30)

	Total (3893)	
	N	(% or SD)
*Income*		
≤ 15.000	424	(10.90)
15.001–25.000	491	(12.60)
25.001–35.000	417	(10.70)
35.001–50.000	438	(11.30)
>50.001	1316	(33.80)
Missing	807	(20.70)
*Region of residence*		
North-West	1460	(37.50)
North-East	535	(13.70)
Centre	471	(12.10)
South	1106	(28.40)
Islands	276	(7.09)
Missing	45	(1.20)
*Tourism dependency*		
Not at all	2066	(53.10)
Slightly	466	(12.00)
Moderately	699	(18.00)
Considerably	270	(6.90)
Extremely	357	(9.20)
*Missing*	35	(0.90)

### Data analysis

A structural model which includes 8 constructs and 7 proposed relationships was built and analyzed with a partial least square structural equation modeling (PLS-SEM) approach, guided by Hair, Hult, Ringle, and Sarstedt (2017). This approach was used due to the complexity of the model which includes both reflective and formative constructs as well as the exploratory nature of this study (do Valle and Assaker, 2016; Hair et al., 2014; Hair et al., 2017). A repeated indicators approach with 2 steps was followed for the two reflective – formative constructs of Vaccine Concerns and Travel Intention (Chin 1998; Duarte and Amaro 2018; Hair et al., 2017). The outer loadings, composite reliability, and the average variance extracted (AVE) were used to establish the reliability and validity of reflective constructs; while the weights of all lower-order components and multicollinearity issues were examined for formative constructs (Hair et al., 2017). Afterward, the path coefficients, the coefficient of determination (R^2^ value), and the effect size (f^2^ and Q^2^) were observed to estimate the proposed relationships as well as the predictive power of the model and the independent variables. The data analysis was conducted using SmartPLS 3.0.

## Findings

### The measurement model

The reliability and validity of the reflective constructs, including the lower order components of COVID-19 Vaccine Concerns and Travel Intentions were first evaluated. 5 measurement items of Efficacy Concerns, Safety Concerns, COVID-19 Vaccine Confidence, and Social Media Intensity were eliminated due to the low factor loadings of less than 0.5, which contributed to low values of CR and AVE. The results of the remaining measurement items are presented in Table 2. Most of the outer loadings were above the acceptable value of 0.708 (Hair et al., 2017). Although some of the items had lower factor loadings, the deletion of these items did not result in higher CR and AVE values, hence they were kept. This approach, where loadings of less than 0.70 are often obtained, is not an exception in the social sciences (Hulland, 1999). The values of Cronbach’s α, CR, and AVE were above the thresholds of 0.7, 0.7, and 0.5, respectively (Hair et al., 2017). Additionally, as shown in Appendices 1 and 2, the square root of each lower-order construct’s AVE was higher than its correlation values with other constructs and the Heterotrait-Monotrait ratios of correlations (HTMT) were below the threshold of 0.85, indicating the establishment of discriminant validity (Hair et al., 2017). Next, to validate the two formative higher-order constructs of COVID-19 Vaccine Concerns and Travel Intentions, the weights and significance of the lower-order components were examined. As shown in Table 3, all the weights were well above 0.1 and statistically significant. The variance inflation factor (VIF) values were also well below the recommended value of 5, indicating no issue of multicollinearity of the reflective constructs (Hair et al., 2017). The above statistics signify the validity and reliability of the measurement model.

**Table 2:** The reflective measurement model analysis

	Outer loading	Cronbach's α	CR	AVE
*Efficacy Concerns*		0.841	0.905	0.76
VacConcerns_1	0.886			
VacConcerns_2	0.891			
VacConcerns_5	0.836			
*Safety Concerns*		0.814	0.872	0.582
VacConcerns_4	0.843			
VacConcerns_6	0.859			
VacConcerns_8	0.566			
VacConcerns_9	0.812			
VacConcerns_10	0.694			
*COVID-19 vaccine confidence*	0.858	0.892	0.551	
VacConfidence_1	0.889			
VacConfidence_2	0.885			
VacConfidence_3	0.622			
VacConfidence_4r	0.541			
VacConfidence_5	0.641			
VacConfidence_6	0.892			
VacConfidence_8r	0.632			
*Social media intensity*		0.816	0.841	0.575
SMIntensity_2	0.637			
SMIntensity_4	0.716			
SMIntensity_5	0.710			
SMIntensity_6	0.935			
Travel Craving		0.941	0.955	0.809
TraCraving_1	0.835			
TraCraving_2	0.917			
TraCraving_3	0.930			
TraCraving_4	0.891			
TraCraving_5	0.920			
*Domestic travel intention*		0.852	0.911	0.774
TravelDom_1	0.896			
TravelDom_2	0.916			
TravelDom_3	0.824			
*International travel intention*	0.906	0.941	0.842	
TravelIntl_1	0.922			
TravelIntl_2	0.943			
TravelIntl_3	0.887			
COVID-19 misinformation	1	1	1	1
Tourism dependency	1	1	1	1
COVID-19 vaccination intention	1	1	1	1

**Table 3:** The formative higher-order measurement model analysis

	Weights	T Statistics	95% BCa confidence intervals	VIF
*COVID-19 vaccine concerns*				
Efficacy concerns	0.483	106.296	0.475 – 0.492	1.582
Safety concerns	0.630	122.988	0.620–0.640	1.582
*Travel intention*				
Domestic travel intention	0.557	164.943	0.551 – 0.564	1.262
International travel intention	0.614	142.214	0.606 – 0.623	1.262

### The structural model

**Fig. 1:**
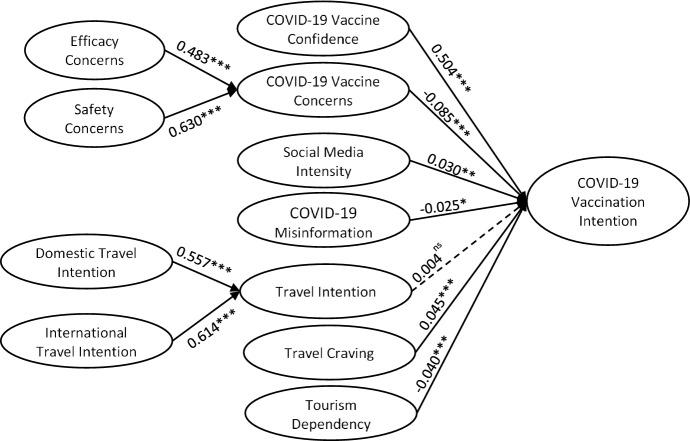
The structural model

Having the measurement model validated in the previous stage, the proposed paths, i.e. the hypotheses, were examined using the path coefficients and t statistics. The results, as shown in Table 4 and Figure 1, indicated that COVID-19 Vaccine Confidence (β = 0.504; t = 21.813), Travel Craving (β = 0.045; t = 3.189) were found to be significantly and positively associated with COVID-19 Vaccination Intention, suggesting support for H1 and H6. Vaccine Concerns (β = -0.085; t = 4.399) and COVID-19 Misinformation (β = -0.025; t = 1.943) were indicated to significantly and negatively influenced COVID-19 Vaccination Intention, supporting H2 and H4. Meanwhile, the results indicated that Travel Intention did not significantly influence COVID-19 Vaccination Intention (β = 0.004.; t = 0.318), rejecting H5. Surprisingly, while Social Media Intensity (β = 0.030; t = 2.461) and Tourism Dependency (β = -0.040; t = 3.040) were found to significantly influence COVID-19 Vaccination Intention, the relationships were opposite to the hypotheses, suggesting a rejection to H3 and H7.

**Table 4:** Path analysis and effect size

Hypotheses/paths		Path coefficients	T Statistics	f^2^
H1	COVID-19 Vaccine Confidence -> COVID-19 Vaccination Intention	0.504	21.813	0.191
H2	COVID-19 Vaccine Concerns -> COVID-19 Vaccination Intention	− 0.085	4.399	0.006
H3	Social Media Intensity -> Vaccination Intention	0.030	2.461	0.001
H4	COVID-19 Misinformation -> COVID-19 Vaccination Intention	-0.025	1.943	0.001
H5	Travel Intention -> COVID-19 Vaccination Intention	0.004	0.318	0.000
H6	Travel Craving -> COVID-19 Vaccination Intention	0.045	3.189	0.003
H7	Tourism Dependency -> COVID-19 Vaccination Intention	− 0.040	3.040	0.002
		R^2^	Q^2^	
		0.348	0.342	

		R^2^	Q^2^	
		0.348	0.342	

^ns^non-significant; ***p<0.01; **p<0.05; *p<0.1

Additionally, R^2^, f^2^, and Q^2^ were considered to establish the predictive power of the independent variables on COVID-19 Vaccination Intention. Overall, with a R^2^ value of 0.348, the structural model was indicated to explain 34.8% of the variability of COVID-19 Vaccination Intention. A Q^2^ value of 0.342 also signified the predictive relevance of this model (Hair et al., 2017). Among the independent variables, COVID-19 Vaccine Confidence had the strongest influence on Vaccination Intention (β = 0.504; t = 21.813) and had a medium effect size (f^2^=0.191). While COVID-19 Vaccine Concerns, Social Media Intensity, COVID-19 Misinformation, Travel Craving, and Tourism Dependency were found to significantly influence COVID-19 Vaccination Intention, their effect size f^2^ values of less than 0.01 indicated rather small effects on COVID-19 Vaccination Intention.

## Discussion and Conclusion

This study took the case of Italy to empirically test social marketing-related factors which are likely to increase COVID-19-vaccination intention, namely COVID-19 vaccine confidence, COVID-19 vaccine concerns, social media intensity, social media misinformation, travel intention, travel craving, and tourism dependency.

The proposed and tested model shows that a majority of our hypotheses are supported and there is a good predictive power of the model in general. Particularly, almost all proposed factors above, accept travel intention, were found to significantly influence COVID-19 vaccination intention. Earlier, Evans and French (2021) suggested that social marketing campaigns for COVID-19 vaccines should (1) educate the public to increase knowledge in safety and efficacy, reduce hesitance, and (2) create a vaccine uptake behavior by creating positive social norms. Nowak et al. (2015) had made similar suggestions for social marketing campaigns addressing general vaccine hesitancy. The proposed and tested model can give some more insights on how these factors should be implemented through social marketing campaigns in a current pandemic context and offers theoretical contributions and social marketing implications as follows.

In terms of theoretical implications, the study makes several contributions. First, both hypotheses related to vaccine concerns (having a negative influence on intention) and vaccine confidence (having a positive influence on intention) have been confirmed. This is in line with past studies underlining the importance of vaccine confidence (e.g., Larson, 2018; Larson, Cooper, Eskola, Katz, & Ratzan, 2011; Larson et al., 2016) in uptaking a vaccine and safety and efficacy being relevant concerns (e.g., Bish et al., 2011; Yaqub et al., 2014). These findings strongly suggest that residents adhere to a social exchange cost-benefit analysis when considering whether to uptake a COVID-19 vaccine, particularly in terms of how safe and efficient they are. However, the only predictor we empirically found to have medium predictive power (while others have low predictive power) on COVID-19 vaccination intention is vaccine confidence. Our measurement of vaccine confidence includes a more varied spectrum of items, including personal health, community health, effectiveness, benefits, risk evaluation, the trustworthiness of the vaccine program, personal protection, and side effects. This goes beyond the two concern factors of safety and efficacy and suggests a wider individual cost-benefit analysis before vaccine uptake.

Second, misinformation was found to have a negative influence on vaccination intention, as has been suggested in past studies (e.g., Fedeli, 2020; Ruiz & Bell, 2021; Williams et al., 2020). This suggests that social representations might also play a role in vaccine uptake, referring to information obtained through social interactions rather than through personal experience. Ruiz and Bell (2021) in particular, found that individuals who would get their news from social media were less likely to get vaccinated for COVID-19 in the USA. In the Italian context, however, our findings show some important differences between exposure to misinformation and social media use in general. Although we hypothesized that social media intensity is likely to influence COVID-19 vaccination intention negatively, we found that this hypothesis is not supported. On the contrary, there was an inverse relationship with social media intensity influencing vaccination intention positively. Our study thus shows that vaccine uptake is likely to be influenced by social representations within certain communities, but that these are not necessarily related to misinformation. On the contrary, based on our findings it can be assumed that exposure to favorable attitudes within a certain community might promote vaccine uptake, while exposure to negative misinformation or the inability to verify the legitimacy of sources might have the opposite effect.

Finally, it has also been hypothesized that individual factors are likely to influence vaccination intention among the wider population (e.g., Detoc et al., 2020; Kwok et al., 2021; Soares et al., 2021). We chose dependency on tourism as a possible predictor, as the tourism industry was particularly hard hit by the pandemic (Wassler & Fan, 2021). More specifically, we chose financial dependency as an essential social exchange factor, while undertaking travel is a non-essential one. In terms of theoretical contributions, we found that financial dependency on the industry does not necessarily lead to a willingness to undertake the vaccine. We originally hypothesized that people with a higher dependency on tourism would be more willing to uptake the vaccine, as this would allow the industry to restart. This was not supported and on the contrary, showed an inverse relationship. The reasons, therefore, are not clear from our data, but it can be assumed that other demographic factors, such as lower levels of education (frequent in the tourism sector as a service industry), might be a potential cause. This is of heightened importance, showing that a cost-benefit analysis by the individual is likely not enough to explain vaccine-related behaviors. On the other hand, in the same context, we also hypothesized that the intention to travel might influence COVID-19 vaccine uptake positively. We assume a similar relationship for travel craving, which is “a travel-focused cognitive-emotional event with aversive or incentive properties experienced when a person who wishes to travel cannot do so, for reasons beyond their control” (Mitev & Irimiás, 2021, p. 2). Our findings show that travel intentions do not influence vaccination intentions positively, while travel craving does. In terms of theory contribution, this shows the predictive power of emotional factors, at times even over immediate rational needs.

This has various implications for social marketing campaigns. The findings suggest that social marketing campaigns should predominantly inform the public about the benefits and risks of undertaking a COVID-19 vaccine to increase vaccine confidence. Topics that should be tackled and communicated include not only safety and efficacy, but also personal health, community health, effectiveness, benefits, risk evaluation, the trustworthiness of the vaccine program, personal protection, and side effects among others. This gains even more importance when these findings are related to our data on misinformation and social media use.

Evans and French’s (2021) hypotheses that a successful COVID-19 social marketing program should guarantee transparency and access to information on a wider range of issues for the general population. This will not help only to limit negative misinformation, but also build trust on a wider range (Ruiz & Bell, 2021). While we are not directly measuring perceived transparency in this study, many of the previously discussed risks and benefits of the vaccines should be communicated to inform the wider population. Misinformation, on the other hand, is often socially spread and counters the official narratives on vaccines. We found this to be particularly critical when the reliability of sources can not be assessed. While we found that social media use can enhance the willingness to undertake vaccines if reliable information can be obtained, social marketing campaigns should not only inform but underline the reliability and provenience of the information provided.

Arguably and most worryingly, the social marketing implications of our findings in the tourism context suggest that it should not be taken for granted that subsections of the population which economically depend on sectors heavily hit by the pandemic will be more willing to undertake the vaccine. On the contrary social marketing efforts should target these important subsectors of the population to heighten their vaccination intentions as soon as possible. When related to travel as a non-essential activity, our findings show that travel intentions do not influence vaccination intentions positively, while travel craving does. In social marketing terms, this simply shows that in times of uncertainty, marketing upcoming trips to citizens would most likely not be effective. On the other hand, instigating travel desire through messages based on craving travel is likely to motivate vaccine uptake. This is not necessarily a message to undertake an actual trip (Mitev & Irimiás, 2021), but rather a reminder of how important the possibility to travel is and the sensations this can stimulate. This is not only a psychological tool to cope with the restrictions caused by the pandemic (Drummond, 2001), but instigating travel desire through social marketing can be a helpful tool for the vaccination campaign as it might encourage vaccine uptake. In additon to encouraging initial vaccination, Travel desire may also be of value in encouraging the maintenance of vaccination coverage via booster shots. Since vaccination protection can wane over time, the infulence of travel desire in encouraging repeat vaccinations can be explored in future research.

Besides its theoretical and managerial implications, this study is not free of limitations. Firstly, as this study adopted the misinformation measure from Williams et. al (2021), it may not fully capture the nuances of the misinformation phenomenon, ie. both positive and negative aspects, suggesting that a more comprehensive measure for recognizing the exposure to (negative) misinformation may be employed as in the study of Pennicook and Rand (2018). Secondly, the study used a sample that remains, despite its relatively large size, highly site-specific (i.e., Italy) and not representative of the overall population under investigation (i.e., Italians having the potential right and possibility to uptake COVID-19 vaccine). These circumstances render the findings hardly generalizable to the overall Italian population and even less outside the Italian context. This said, future studies might be conducted in other countries to cross-culturally validate our findings Furthermore, we actively measure COVID-19 vaccination intentions and not vaccine uptake. The gap between intention to act and actual behavior has been widely studied and needs to be acknowledged by this research. This study also opted to conduct a non-probability sampling approach. The authors strived to increase the diversity of the sample by combining different non-probability sampling techniques and platforms, to allow for the diversity of the initial set of respondents (Kirchherr & Charles, 2018; Morgan, 2008). Nonetheless, this approach might still have biased the study sample and potentially have excluded important segments of the population in terms of the aforementioned demographics.

## Appendix

See Table

**Table 5:** The measurement items

	Measurement items
*Efficacy Concerns*	
VacConcerns_1	I do not trust the COVID-19-vaccine to protect me from the disease while traveling abroad effectively
VacConcerns_2	I am not confident in the COVID-19-vaccine helping me stay healthy while abroad
VacConcerns_5	I am not sure that the Covid19 vaccine will guarantee my travel safety
Safety Concerns	
VacConcerns_4	I worry about the long-term effects of the COVID-19-vaccine on my health
VacConcerns_6	I worry about the side effects of the COVID-19-vaccine
VacConcerns_8	I fear the injection when taking the COVID-19-vaccine because of the pains
VacConcerns_9	I worry that the side effects of the COVID-19-vaccine while abroad can decrease my enjoyment of the holiday experience
VacConcerns_10	I fear that I may not readily get medical assistance if experiencing the side effects of the COVID-19-vaccine while abroad
*COVID-19 Vaccine Confidence*	
VacConfidence_1	The COVID-19-vaccine is important for my health
VacConfidence_2	Having the COVID-19-vaccine is important for the health of others in my community
VacConfidence_3	The COVID-19-vaccine offered by the government program in my community is effective and beneficial
VacConfidence_4r	The COVID-19-vaccine carries more risks than older vaccines (reversed)
VacConfidence_5	The information I receive about the COVID-19-vaccine from the vaccine program is reliable and trustworthy
VacConfidence_6	Getting the COVID-19-vaccine is a good way to protect myself from disease
VacConfidence_8r	I am concerned about the serious adverse effects of the COVID-19-vaccine (reversed)
*Social Media Intensity*	
SMIntensity_2	I am proud to tell people I’m on Social Media
SMIntensity_4	I feel out of touch when I haven’t logged onto Social Media or a while
SMIntensity_5	I feel I am part of the Social Media community
SMIntensity_6	I would be sorry if Social Media shut down
Travel Craving	
TraCraving_1	Now that vaccines against COVID-19 exist, I’m thinking about travelling more frequently than before
TraCraving_2	Now that vaccines against COVID-19 exist, my craving for travel is stronger than before
TraCraving_3	Now that vaccines against COVID-19 exist, I’m spending more time thinking about travelling than before
TraCraving_4	Now that vaccines against COVID-19 exist, it is more difficult to resist travelling than before
TraCraving_5	Now that vaccines against COVID-19 exist, my overall travel craving is higher than before
*Domestic Travel Intention*	
TravelDom_1	I intend to travel within Italy in the next 12 months
TravelDom_2	It is likely that I will travel within Italy in the next 12 months
TravelDom_3	I plan to travel within Italy in the next 12 months
*International Travel Intention*	
TravelIntl_1	I intend to travel abroad in the next 12 months
TravelIntl_2	It is likely that I will travel abroad in the next 12 months
TravelIntl_3	I plan to travel abroad in the next 12 months
COVID-19 Misinformation	How much information have you seen or heard about COVID-19 that seemed fake or made up?
Tourism Dependency	How much does your income depend on tourism?
COVID-19 Vaccination Intention	Please indicate the likelihood of you getting the COVID-19 vaccine

5.
